# Diverse Regulation of Temperature Sensation by Trimeric G-Protein Signaling in *Caenorhabditis elegans*

**DOI:** 10.1371/journal.pone.0165518

**Published:** 2016-10-27

**Authors:** Tomoyo Ujisawa, Akane Ohta, Misato Uda-Yagi, Atsushi Kuhara

**Affiliations:** 1 Laboratory of Molecular and Cellular Regulation, Graduate school of Natural Sciencey, Konan University, 8-9-1 Okamoto, Higashinada-ku, Kobe 658–8501, Japan; 2 Laboratory of Molecular and Cellular Regulation, Faculty of Science and Engineering, Konan University, 8-9-1 Okamoto, Higashinada-ku, Kobe 658–8501, Japan; 3 Institute for Integrative Neurobiology, Konan University, 8-9-1 Okamoto, Higashinada-ku, Kobe 658–8501, Japan; East Carolina University, UNITED STATES

## Abstract

Temperature sensation by the nervous system is essential for life and proliferation of animals. The molecular-physiological mechanisms underlying temperature signaling have not been fully elucidated. We show here that diverse regulatory machinery underlies temperature sensation through trimeric G-protein signaling in the nematode *Caenorhabditis elegans*. Molecular-genetic studies demonstrated that cold tolerance is regulated by additive functions of three Gα proteins in a temperature-sensing neuron, ASJ, which is also known to be a light-sensing neuron. Optical recording of calcium concentration in ASJ upon temperature-changes demonstrated that three Gα proteins act in different aspects of temperature signaling. Calcium concentration changes in ASJ upon temperature change were unexpectedly decreased in a mutant defective in phosphodiesterase, which is well known as a negative regulator of calcium increase. Together, these data demonstrate commonalities and differences in the molecular components concerned with light and temperature signaling in a single sensory neuron.

## Introduction

Temperature sensation is essential for the life of living organisms, including humans, because temperature affects the rate of biochemical reactions, and, temperature change provides an important cue in the regulation of metabolic processes. Temperature sensation is performed by a variety of temperature-sensing molecules. Heat-shock proteins are key components of the stress response of individual cells. In the nervous system, ambient temperature information is received by a temperature receptor on the plasma membrane. Transient receptor potential (TRP) channels are well known as temperature receptors [1]. Several types of TRP channels cooperate to receive a wide range of temperatures, e.g., TRPV1 receives high temperatures and TRPA1 receives cool temperatures [1]. TRP-independent temperature-sensing mechanisms have also been identified. In *Drosophila*, the light-receptor protein, rhodopsin, plays a role in temperature sensation, with downstream trimeric G protein signaling [2]. In contrast, in the nematode *Caenorhabditis elegans*, the light-receptor protein LITE-1 in the temperature-sensing neuron is not involved in temperature sensation [3], although trimeric G protein-coupled signaling is required for the temperature response at the individual level [3,4].

*C*. *elegans* is a good model animal for studying sensory systems, because it has a simple nervous system composed of only 302 neurons, and it has been well-studied using powerful molecular genetic approaches. Temperature responses by *C*. *elegans* have been analyzed with respect to several phenomena, including dauer larva formation, thermotactic behavior and cold tolerance [3–6].

Temperature sensation by *C*. *elegans* has been well analyzed using thermotactic behavior [6]. The AFD sensory neuron is a major temperature-sensing component of thermotaxis [6,7]. The AWC sensory neuron also responds to temperature changes [4] but whether AWC is primarily a temperature-sensing neuron is unclear [8]. In the AFD neuron, temperature sensation is regulated by three receptor-type guanylyl cyclases (GCs), phosphodiesterase (PDE) and the cyclic-nucleotide-gated channel (CNG), TAX-4/TAX-2/CNG-3 [8]. A PDE regulates temperature adaptation of the AFD neuron [9]. However, the trimeric G protein α subunit (Gα) underlying temperature signaling in the AFD temperature-sensing neuron has not been identified. A recent study reported that ectopic expression of GCs can confer thermosensitivity to heterologous cells, implying that the GCs in AFD may in fact function as the thermosensor [10]. Similar sequences of cGMP-dependent molecular events are displayed in vertebrate photoreceptors and olfactory neurons [11–16], although light and odorants are received by a G-protein-coupled seven-transmembrane receptor (GPCR) in these sensory neurons.

**Fig 1:**
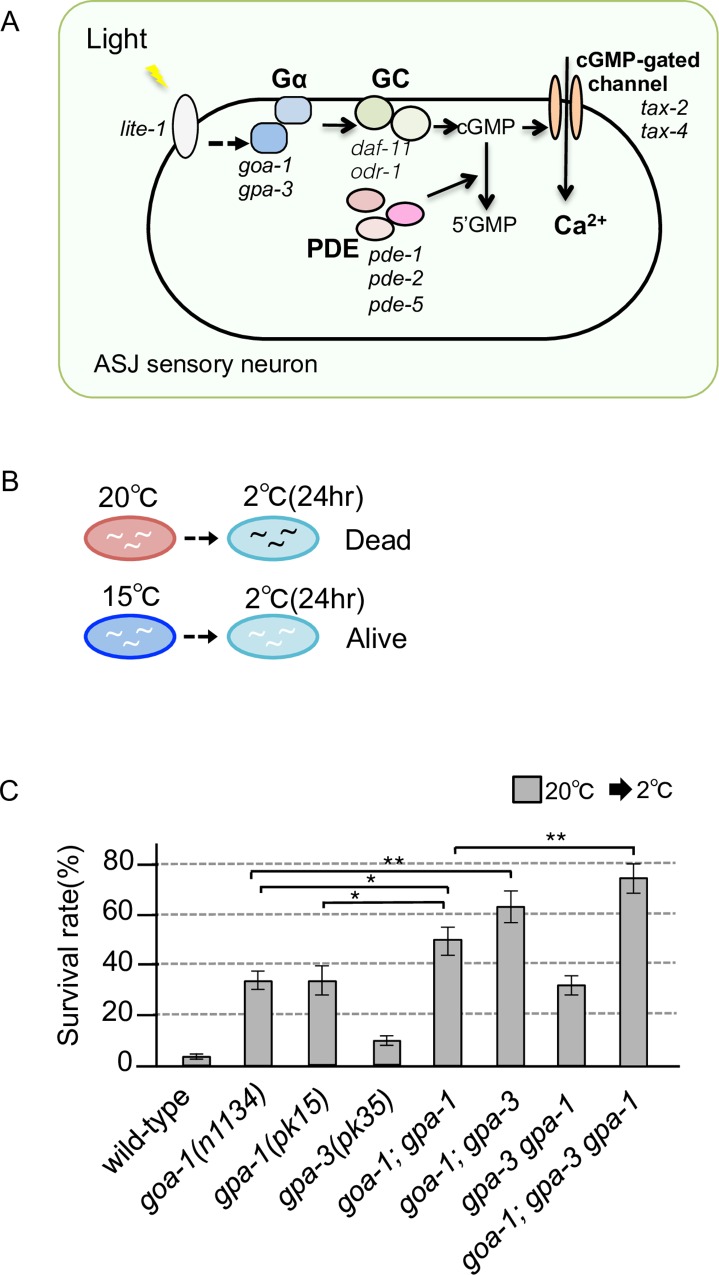
Genetic redundancy of Gα protein in cold tolerance. (A) Molecular model for the G-protein-mediated pathway of light sensation by the ASJ sensory neuron [19]. Gα, a trimeric G-protein α subunit (*goa-1* or *gpa-3*), GC, guanylyl cyclase (*daf-11* or *odr-1*), cGMP, cyclic guanosine monophosphate, PDE, phosphodiesterase (*pde-1*, *pde-2* or *pde-5*). (B) Schema indicating cold tolerance of *C*. *elegans*. Wild-type animals died after a cold stimulus of 2°C for 24 hours, when they were cultivated at 20°C. In contrast, wild-type animals cultivated at 15°C survived. (C) Cold-tolerance survival phenotypes of Gα mutants. Full allele names of the double and triple mutants are the same as those of the single mutants. For each assay, n ≥ 12. Analysis of variance followed by Dunnett's *post-hoc* test was used for multiple comparisons. *P < 0.05; **P < 0.01.

In the cold-tolerance phenomenon exhibited by *C*. *elegans*, temperature information is received by a pair of head sensory neurons, ASJs, in which a cGMP-gated channel TAX-4/TAX-2 is essential for temperature signaling [3,17,18]. In addition to temperature, the ASJ sensory neurons also receive light and pheromones [5,19,20]. The molecular mechanism underlying light-sensory signaling in ASJ has previously been determined in detail. Multiple Gα-, GC-, and PDE-proteins function in a coordinated sequence (Fig 1A) [19]. Light is received by a photoreceptor LITE-1 in ASJ, which sequentially activates Gα (GOA-1 and GPA-3), GC (DAF-11 and ODR-1) and a CNG channel TAX-4/TAX-2; PDE (PDE-1, PDE-2 and PDE-5) acts as a negative regulator of light signaling by hydrolyzing cGMP to 5′GMP [19]. With the exception of the CNG channel, TAX-4/TAX-2, the molecular components of temperature signaling in ASJ are not well understood [3]. Mutant animals impairing TAX-4/TAX-2 showed abnormal enhancement of cold tolerance [3]. Similar abnormalities were observed in mutants defective in Gαs, GCs and PDEs, which are involved in light-signal transduction. Although the mutants defective in these molecules showed a similar abnormality of cold tolerance as the *tax-4* and *tax-2* mutants [3], the physiological differences in the ASJ sensory neuron of these mutants defective in G-protein signaling have not been identified. We therefore analyzed in detail the roles of these molecules in the physiology of ASJ temperature signaling.

In this study, we investigated the commonalities and differences in molecular components between light- and temperature-signaling in the ASJ sensory neuron. Three Gα proteins are present at the sensory ending of the dendrite of the ASJ neuron. Specific expression analysis showed that these Gα proteins regulate cold tolerance in ASJ. Calcium imaging demonstrated that three Gα proteins are additively involved in neuronal activation of ASJ. Although PDE is a negative regulator of light signaling in ASJ, four mutations of the PDEs decreased neuronal activity. This result is consistent with physiological observations on vertebrate photoreceptor neurons, in which strong calcium influx into sensory neurons with defective PDE down-regulates GC [21,22], resulting in inactivation of the neuron. Together these observations, demonstrate that trimeric G-protein-mediated signaling is required for temperature sensation.

## Materials and Methods

### Strains and Genetics

We used the following *C*. *elegans* strains: wild-type N2 Bristol, MT2426 *goa-1(n1134)*, NL332 *gpa-1(pk15)*, NL335 *gpa-3(pk35)*, *goa-1(n1134); gpa-1(pk15)*, DR47 *daf-11(m47)*, CX2336 *odr-1(n1936)*, CX2454 *daf-11(sa195); odr-1(n1936)*, IK616 *pde-1(nj57)*, IK618 *pde-2(nj58)*, *pde-3(nj59)*, IK510 *pde-5(nj49)*, IK798 *pde-1(nj57) pde-5(nj49); pde-2(nj58)*, TQ1828 *pde-1(nj57) pde-5(nj49); pde-3(nj59); pde-2(tm3098)*, All mutations used in this study were null mutations [19,23–25].

The multiple mutants (KHR80 *goa-1(n1134); gpa-3(pk35)*, KHR81 *gpa-3(pk35) gpa-1(pk15)* and KHR82 *goa-1(n1134); gpa-3(pk35) gpa-1(pk15)*, KHR88 *pde-3(nj59); gpa-1(pk15); Ex[trx-1p*::*yc3*.*60*, *ges-1p*::*taqRFP*, *rol-6gf]*, KHR89 *pde-5(nj49); gpa-1(pk15); Ex[trx-1p*::*yc3*.*60*, *ges-1p*::*taqRFP*, *rol-6gf]*) were constructed using standard genetic methods and verified by sequencing their mutation sites.

### Statistical Analysis

All error bars in the figures indicate the standard error of the mean (SEM). Statistical analyses shown in all figures were performed by one-way analysis of variance (ANOVA), followed by Dunnett’s *post hoc* tests for multiple comparisons. Single asterisks (*) and double asterisks (**) in the figures indicate *p* < 0.05 and *p* < 0.01, respectively.

### Molecular Biology

The plasmids *trx-1p*::*goa-1 cDNA(*pUDA3), *trx-1p*::*gpa-1 cDNA*(pUDA4) and *trx-1p*::*gpa-3 cDNA*(pUDA5) contain *goa-1 cDNA* (GenBank: AY008140.1), *gpa-1* cDNA (GenBank: AY008124.1), *gpa-3 cDNA* (GenBank: AY008126.1), respectively, that were PCR amplified from the N2 genome and the 3′-UTR of *unc-54*. Additionally, these plasmids included the ASJ-specific *trx-1* promoter (1.0 kb) from pQZ34 *trx-1p*::*GFP* (a gift from Dr. Alcedo). *gcy-5p*::*goa-1 cDNA*(pUDA10), *gcy-5p*::*gpa-1 cDNA*(pUDA11) and *gcy-5p*::*gpa-3 cDNA*(pUDA12) contained the ASER specific *gcy-5* promoter from *pGcy-5p*::*mCherry* (a gift from Dr. Iino). *goa-1p*::*goa-1 cDNA*(pTOM053) plasmid included *goa-1*’s own promoter (3.0 kb upstream of C26C6.2) from PCR amplification. *gpa-1p*::*gpa-1* cDNA(pTOM54) plasmid included *gpa-1*’s own promoter (1.5 kb upstream of T19C4.6a) from PCR amplification. *gpa-3p*::*gpa-3* cDNA(pTOM55) plasmid included *gpa-3*’s own promoter (3.0 kb upstream of E02C12.5a) from PCR amplification.

To construct GPA-1 fused with fluorescent protein [*trx-1p*::*gpa-1cDNA*::*venus* (pUDA14)] for localization analysis, we deleted the stop codon of *gpa-1 cDNA* by site-directed mutagenesis and then the cDNA was inserted between the *trx-1* promoter and the *venus* of *trx-1p*::*daf-28*::*venus* (pOTA10), replacing *daf-28* in the DNA region. The *trx-1p*::*gpa-3cDNA*::*venus* (pUDA15) and the *trx-1p*::*goa-1cDNA*::*venus* (pUDA13) were made using similar strategies.

### Cold-tolerance Assay

The cold-tolerance assay was performed as described previously [3,26,27]. In the cold-tolerance assay, we used uncrowded and well-fed young adult animals when they commenced to lay eggs. One animal was placed on a 3.5-cm plate containing 6 mL of nematode growth medium (NGM) with 2% (w/v) agar, on which *Escherichia coli* OP50 was seeded; the adult animals were removed after 8–12 h; and the progeny were cultivated for 85–90 h at 20°C. Approximately 70–150 animals were placed on a plate. The plates containing uncrowded and well-fed animals, at fresh adult stage when they started to lay eggs, were transferred to 2°C in a refrigerated cabinet (CRB-41A Hitachi, Japan). Temperature in the refrigerated cabinet was monitored by both a digital thermometer and a mercury thermometer. After 24 h, the plates were transferred to 15°C overnight and the living and dead animals on the plate were counted.

### *In vivo* Calcium Imaging

*In vivo* calcium imaging of the ASJ sensory neuron was performed essentially according to previous reports [3,28]. Worms expressing yellow cameleon 3.60 driven by the *trx-1* promoter, *trx-1p*::*yc3*.*60* (pTOM13) were used for *in vivo* calcium imaging of the ASJ neuron. Animals were glued onto a 2% (w/v) agar pad on glass and immersed in M9 buffer under a cover-glass. Sample preparation was completed within 3 min. The sample was then placed onto a Peltier-based thermocontroller (Tokai Hit Co. Ltd., Fujinomiya, Japan) on the stage of an Olympus IX81 microscope (Olympus Corporation, Tokyo, Japan) at the initial imaging temperature for about 2 min, and fluorescence was observed using a Dual-View (Molecular Devices, USA) optical system. Fluorescence images of donor and acceptor fluorescent protein in yellow cameleon were simultaneously captured using an EM-CCD camera EVOLVE512 (Photometrics, USA). Images were taken with approximately 100-ms exposure times and 1×1 binning. The temperature on the agar pad was monitored by a thermometer system, MATS-5500RA-KY (Tokai Hit). For each imaging experiment, the fluorescence intensities were measured using the MetaMorph (Molecular Devices) image analysis software system. Relative changes in intracellular calcium concentration were measured as the change in the acceptor/donor fluorescence ratio of yellow cameleon protein. All band-pass filters for experiments using yellow cameleon were as described in previous reports [28].

### Germline Transformation

**Fig 2:**
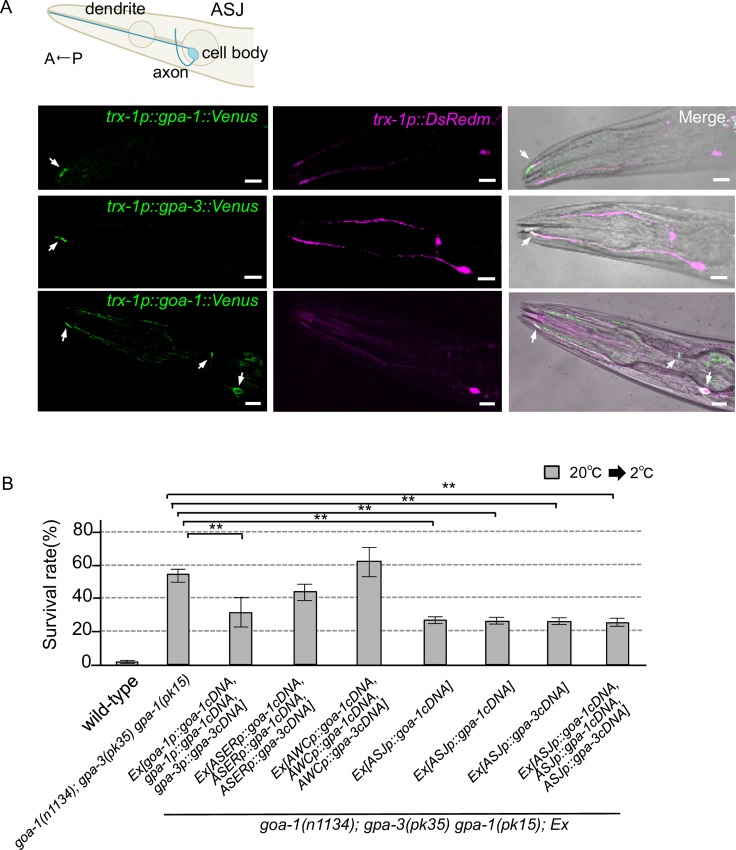
Functional redundancy of Gα at ASJ ciliated endings. (A) Intracellular localization of each Gα protein in the ASJ sensory neuron. The wild type with ASJ-specific expression of dsRedm and each Gα (GPA-1, GPA-3 or GOA-1,)::Venus were analyzed by confocal microscopy. The top panel is a schematic diagram of the ASJ sensory neuron. The left-hand images indicate localization of Gα::Venus in ASJ. The center images indicate dsRedm localization in ASJ. The right-hand panels are merged images of the left and center images and the bright-field image. The arrows in the panels indicate co-localization sites of Gα::Venus and dsRedm in ASJ. Scale bar, 10 μm. (B) Specific expression of Gα genes in the ASJ sensory neuron partially rescues the abnormal cold tolerance of the Gα triple mutant. 20°C-cultivated Gα triple mutants showed abnormal enhancement of cold tolerance, which was partially rescued by expressing individual Gα genes in ASJ. In this figure, we used *goa-1p* for *goa-1*’s own promoter, *gpa-1p for gpa-1*’s own promoter, *gpa-3p for gpa-3*’s own promoter, *gcy-5p* as a promoter for expressing genes in the ASER gustatory neuron, *ceh-36p* as a promoter for expressing genes in the AWC sensory neuron of the thermotaxis neural circuit, and *trx-1p* as a promoter for specifically expressing genes in the ASJ thermosensing neuron. For each assay, n ≥ 10. Error bars indicate standard errors of the means. Analysis of variance followed by Dunnett's *post-hoc* test was used for multiple comparisons. **Significantly different (*P* < 0.01).

**Fig 3:**
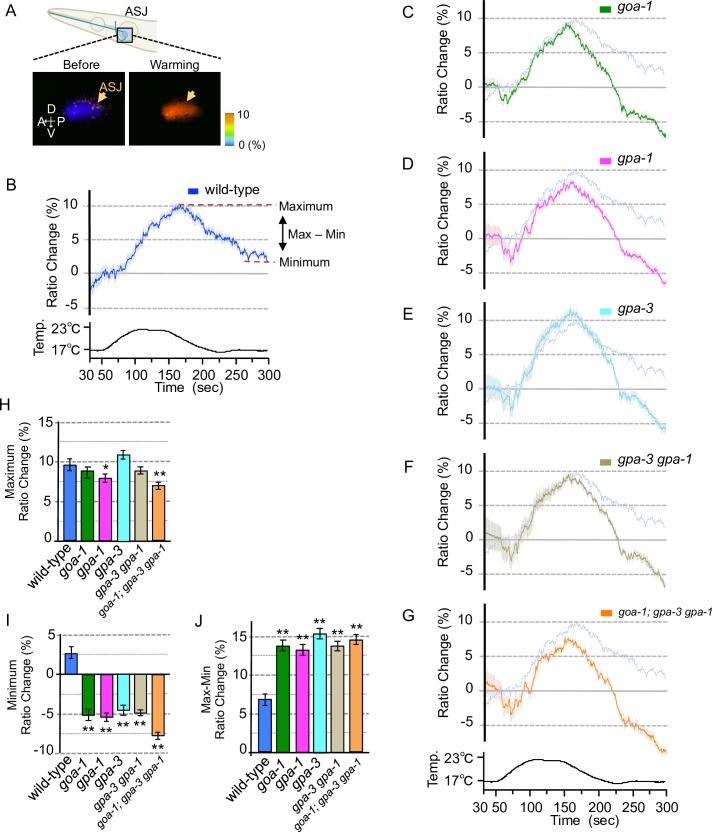
Calcium concentration changes in ASJ of Gα mutants with warming stimuli. (A) Wild type with ASJ specific expression of YC3.60 [*trx-1p*::*yc3*.*60*] used for calcium imaging. The schematic diagram indicates an ASJ sensory neuron in the head. The lower pair of images shows the response to a warming stimulus of the ASJ cell body in the 20°C-cultivated wild type. Arrows indicate ASJ cell body. (B) *In vivo* calcium imaging of ASJ from the wild type cultivated at 20°C. Relative calcium concentrations were measured as the yellow/cyan fluorescence ratio of YC3.60. The calcium concentration in ASJ changed upon the warming stimulus (*n* = 18). (C–G) Calcium imaging of ASJs in Gα mutants cultivated at 20°C. The transgene of *trx-1p*::*yc3*.*60* was introduced into each Gα mutant and we measured relative calcium concentrations under warming stimuli, as in the wild-type experiment (panel B, shown as pale blue lines in panels C–G; these experiments were performed simultaneously; *n* = 18–20). The temperature change during this test is indicated in the bottom chart. (H) The bar chart shows the average ratio changes in the period 5 s before the maximum point to 5 s after the maximum point in the experiments shown in graphs B–G. (I) The bar chart shows the average ratio changes around the minimum point during 10 s from 280 s to 290 s in the experiments shown in graphs B–G. (J) The bar chart shows the differences between the average maximum and minimum values shown in graphs H and I. Error bars indicate SEM (B–J). Analysis of variance followed by Dunnett's *post-hoc* test was used for multiple comparisons (H–J, compared with the wild type). *P < 0.05; **P < 0.01. Colors used in the bar graphs in H–J, are the same as those used for the corresponding response curves in B–G.

**Fig 4:**
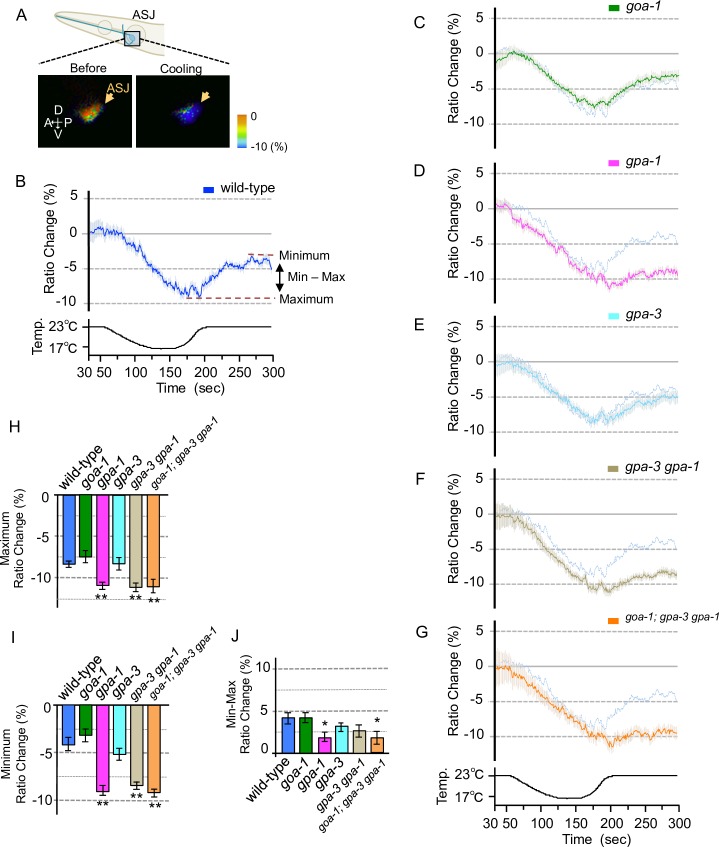
Calcium concentration changes in ASJ of Gα mutants with cooling stimuli. ASJ specifically expressing YC3.60 in wild-type worms and Gα mutants. (A) The schematic diagram indicates an ASJ sensory neuron in the head. The bottom panel shows the response to cooling stimuli of the ASJ cell body in 20°C-cultivated wild type, with a scale showing pseudo-color images depicting the fluorescence ratio of cameleon before and during temperature change. Arrows indicate ASJ cell body. (B) *In vivo* calcium imaging of ASJ from wild-type samples cultivated at 20°C. Relative calcium concentrations were measured as the yellow/cyan fluorescence ratio of YC3.60. The calcium concentration in ASJ changed upon temperature warming stimuli. *n* = 14. (C–G) relative calcium concentrations under cooling stimuli in Gα mutants, as in the wild-type experiment (panel B; shown as pale blue lines in C–G; these experiments were performed simultaneously; n = 18–20). Time and temperature in the experimental field during this test are indicated in the bottom chart. (H) The bar chart shows the average ratio during 5 s before the maximum point to 5 s after the maximum point in graphs B–G. (I) The bar chart shows the average ratio change around the minimum point during 10 s from 280 to 290 s in graphs B–G. (J) The bar chart shows the average ratio change of the difference value between the maximum and minimum points in graphs B–G. Error bars indicate SEM (B–J). Analysis of variance followed by Dunnett’s *post-hoc* test was used for multiple comparisons (H–J, compared with the wild type). *P < 0.05; **P < 0.01. Colors used in the bar graphs in H–J, are the same as those used for the corresponding response curves in B–G.

**Fig 5:**
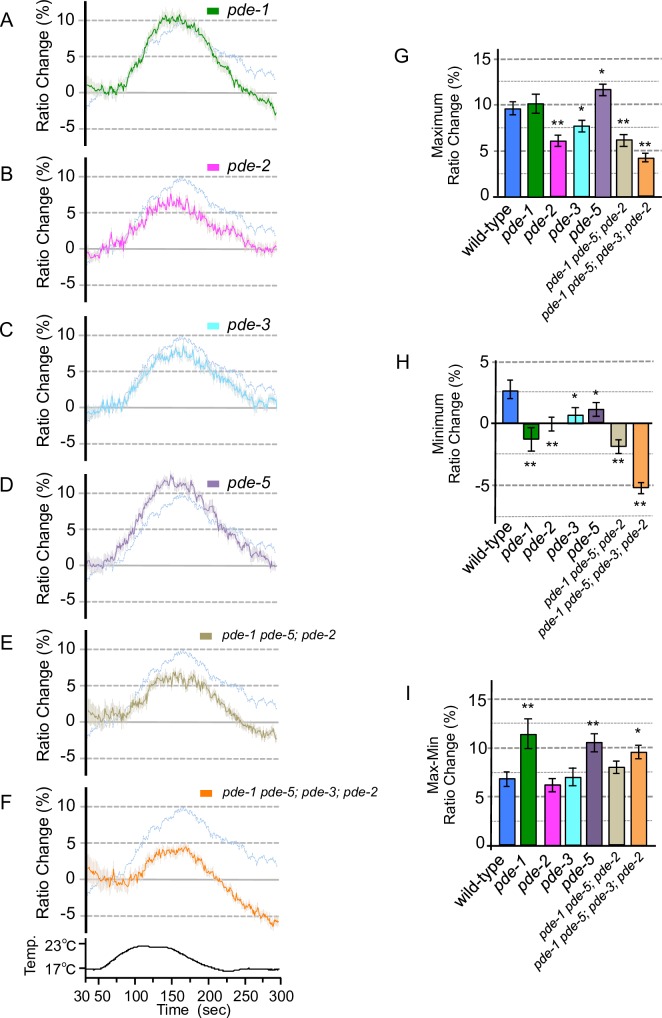
Calcium concentration changes in ASJ of PDE mutants with warming stimuli. (A–F) Calcium imaging of ASJ from PDE mutants cultivated at 20°C. The transgene of *trx-1p*::*yc3*.*60* was introduced into each PDE, mutant and the relative calcium concentration changes under warming stimuli were measured as in the wild-type experiment shown in Fig 3B (pale blue lines in panels A–F.; the experiments were performed simultaneously; *n* = 15–19). The temperature change during the experiment is indicated in the bottom chart. (G) The bar chart shows the average ratio changes from 5 s before the maximum point to 5 s after the maximum point in Figs 3B and 5A–F. (H) The bar chart shows the average ratio changes from around minimum point during 10 s from 280 to 290 s of the experiments shown in Figs 3B and 5A–F. (I) The bar chart shows the average ratio changes of the difference values between maximum and minimum points of the experiments shown in Figs 3B and 5A–F. Error bars indicate standard error of mean (A–I). Analysis of variance followed by Dunnett’s *post-hoc* test was used for multiple comparisons (G–I, compared with the wild type). *P < 0.05; **P < 0.01. Colors used in the bar graphs in G–I, are the same as those used for the corresponding response curves in A–F.

**Fig 6:**
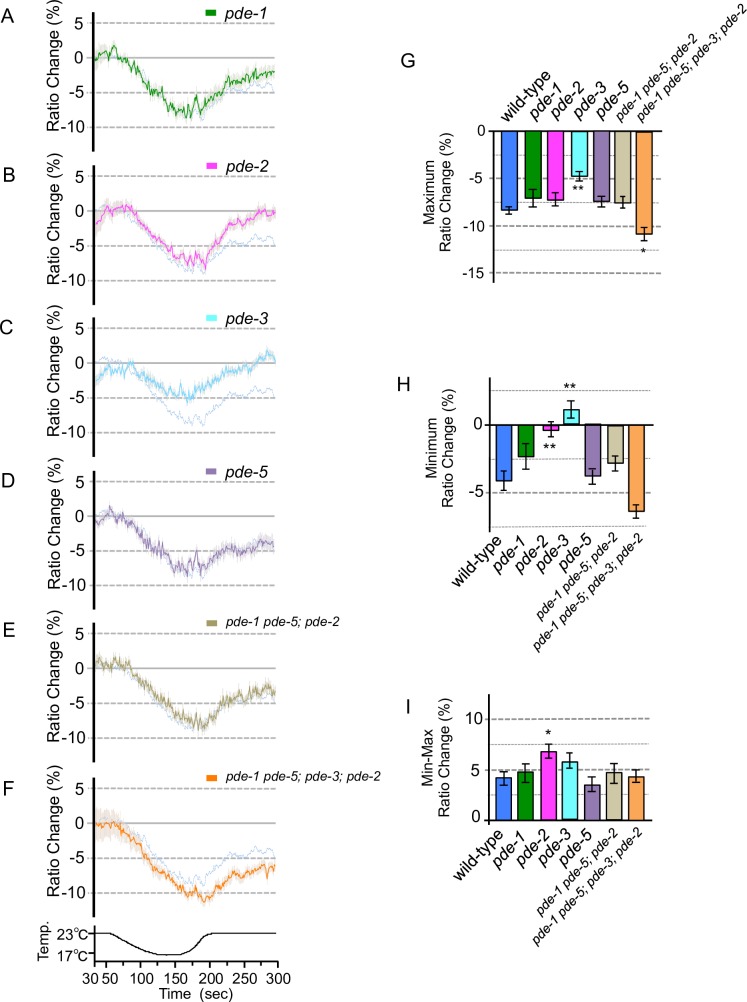
Calcium concentration changes in ASJ of PDE mutants with cooling stimuli. ASJ specifically expressing YC3.60 in wild-type worms and PDE mutants. (A–F) we measured relative calcium concentrations under cooling stimuli as in the wild-type experiment shown in Fig 4B (pale blue lines; these experiments were performed simultaneously; *n* = 13–21). Temperature changes during the experiment are indicated in the bottom chart. (G) The bar chart shows the average ratio changes from 5 s before the maximum point to 5 s after the maximum point in Figs 4B and 6A–F. (H) The bar chart shows the average ratio changes from around minimum point during 10 s from 280 to 290 s of the experiment in Figs 4B and 6A–F. (I) The bar chart shows the average ratio changes of the difference values between maximum and minimum points of the experiment in Figs 4B and 6A–F. Error bars indicate SEM (A–I). Analysis of variance followed by Dunnett’s *post-hoc* test was used for multiple comparisons (G–I, compared with the wild type). *P<0.05; **P < 0.01. Colors used in the bar graphs in G–I, are the same as those used for the corresponding response curves in A–F.

**Fig 7:**
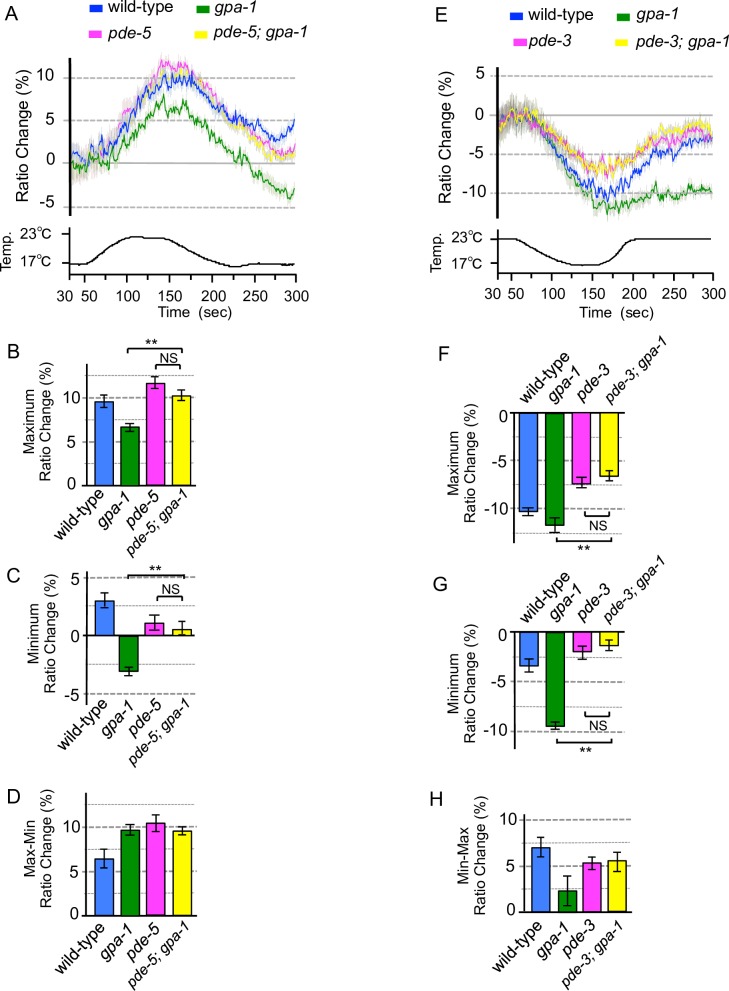
Genetic epistasis between PDE and Gα mutations on temperature signaling. ASJ specifically expressing YC3.60 in wild-type worms and Gα or/and PDE mutants. (A) we measured relative calcium concentrations under warming stimuli. The blue, green, and magenta lines indicate calcium concentration changes in wild-type animals, *gpa-1* and *pde-5* mutants, respectively. The yellow line indicates calcium concentration in the *pde-5; gpa-1* mutants (*n* = 9–14). Temperature changes during the experiment are indicated in the lower chart. (B) The bar chart shows the average ratio changes from 5 s before the maximum point to 5 s after the maximum point of the experiment shown in panel A. (C) The bar chart shows the average ratio changes from around minimum point during 10 s from 280 to 290 s of the experiment shown in panel A. (D) The bar chart shows the average ratio changes of the difference values between maximum and minimum points of the experiment shown in panel A. Colors used in graphs B–D are the same as those used for the corresponding response curves in A. (E) We measured relative calcium concentrations under cooling stimuli. The blue, green, and magenta lines indicate calcium concentration changes in wild-type animals, *gpa-1* and *pde-3* mutants, respectively. The yellow line indicates calcium concentration in the *pde-3; gpa-1* mutants. *n* = 11–13. Temperature changes during the experiment are indicated in the bottom chart. (F) The bar chart shows the average ratio changes from 5 s before the maximum point to 5 s after the maximum point in Fig 7E. (G) The bar chart shows the average ratio change from around minimum point during 10 s from 280 to 290 s of the experiment in Fig 7E. (H) The bar chart shows the average ratio change of the difference value between maximum and minimum points of the experiment in Fig 7E. Colors used in graphs F-H are the same as those used for the corresponding response curves in E. Error bars indicate SEM (A–H). Analysis of variance followed by Dunnett’s *post-hoc* test was used for multiple comparisons. *P < 0.05; **P < 0.01. NS, not significant (*P* > 0.05).

Germline transformations were performed essentially as described previously [29] with co-injection mixes consisting of experimental DNA at various concentrations (5–100 ng/μl) and the transgenic marker, which was pRF4 *rol-6gf* (Fig 2A), pAK62 *AIYp*::*GFP* and pKDK66 *ges-1p*::*NLS-GFP* (Fig 2B), or pRF4 *rol-6gf* and pNAS88 *ges-1p*::*NLS-tagRFP* (Figs 3–7 and S1 Fig).

### Confocal Microscopy

The following procedure was used for preparation of samples: a 2% (w/v) agarose gel on a glass micro-slide was covered with 10 μL of 100 mM NaN_3_, and a few adult worms were placed on the gel. The gel was then covered by glass. Fluorescent images were analyzed by confocal laser microscopy (FV1000-IX81 with GaAsP PMT, Olympus), using FV10-ASW software (Olympus).

## Results and Discussion

### Trimeric G-proteins are additively involved in cold tolerance of *C*. *elegans*

The cold tolerance in *C*. *elegans* depends on their cultivation temperature. 15°C-cultivated animals survive at 2°C, whereas 20°C-cultivated animals do not (Fig 1B) [3]. We previously reported that temperature information for cold tolerance is received by the ASJ sensory neurons, which are also known to be light- and pheromone-sensory neurons. The cGMP-gated channels TAX-2/TAX-4 are essential for transduction of the temperature signal in ASJ sensory neurons [3]. In transduction of the light signal in ASJ, Gα proteins GOA-1 and GPA-3 function redundantly, guanylyl cyclase DAF-11 and ODR-1 function redundantly, and phosphodiesterases PDE-1, -2 and -5 function redundantly (Fig 1A) [19]. Although single mutants defective in either GOA-1, GPA-1 or GPA-3 show similar abnormalities in cold tolerance as *tax-4* and *tax-2* mutants [3], the redundancy or additive role of these three Gαs in cold tolerance and in the ASJ temperature response at the physiological level has not been evaluated. We therefore phenotypically analyzed cold tolerance and ASJ neuronal activity in animals impairing multiple Gα genes.

Each *goa-1* and *gpa-1* single mutant showed an abnormal increase in cold tolerance at 2°C for 24 hours, after cultivation at 20°C, similar to that described previously (Fig 1C, *goa-1*, *gpa-1*). We found that the *goa-1; gpa-1* double mutants showed a stronger abnormality than that in each single mutant (Fig 1C, *goa-1; gpa-1*), implying that these genes work additively. Mutation in *gpa-3*, which is essential for light signaling, did not induce any marked abnormality in cold tolerance (Fig 1C). However, this *gpa-3* mutation enhanced the cold-tolerance effect of *goa-1* (Fig 1C, *goa-1; gpa-3*), and the triple mutant *goa-1; gpa-3 gpa-1* also showed a strong cold-tolerance phenotype (Fig 1C, *goa-1; gpa-3 gpa-1*). These phenotypic analyses suggest that these three Gαs function additively in cold tolerance.

### Gα proteins are present at the sensory ending of the dendrite in ASJ

Genetic analysis revealed that the three Gα proteins, GOA-1, GPA-1 and GPA-3, function additively in cold tolerance (Fig 1C). The temperature stimuli for cold tolerance are at least received by the ASJ sensory neurons, which are also light and pheromone sensors [5,19,20]. The *gpa-1* and *gpa-3* genes are expressed in several sensory neurons, including ASJ, and the *goa-1* gene is expressed in almost all neurons [23]. Because temperature sensation by ASJ is regulated by the cGMP-gated channels TAX-4/TAX-2, which are specifically localized in the sensory endings of the dendrites of sensory neurons [17,18], the temperature stimulus is probably received at the sensory ending of the ASJ neuron. To analyze whether these Gα proteins are involved in temperature sensation in ASJ, we observed whether these Gαs are present at the sensory ending of the ASJ dendrite using the yellow fluorescent protein, Venus.

We expressed *gpa-1*cDNA fused with the *venus* gene (*gpa-1*::*venus*) specifically in the ASJ sensory neuron, by driving with the ASJ-specific promoter, *trx-1p*. We also expressed red fluorescent protein (DsRedm) in ASJ, as an expression marker for GPA-1::Venus. The fluorescence of GPA-1::Venus was observed at the sensory ending of the ASJ dendrite (Fig 2A). A similar localization pattern was observed for GPA-3::Venus (Fig 2A), indicating that both GPA-1 and GPA-3 are located at the sensory ending of ASJ. The fluorescence of GOA-1::Venus was observed in the entire cytoplasm of ASJ, i.e., sensory ending, cell body and axon. These observations indicate that Gα proteins are present at the sensory ending of ASJ.

### Gα genes in ASJ are necessary to regulate cold tolerance

The trimeric G-protein α subunits GOA-1, GPA-1 and GPA-3 are involved in cold tolerance, and they are present at the sensory ending of the temperature-sensing neuron, ASJ. To examine whether the abnormal cold tolerance observed in Gα mutant animals is caused by defective Gα genes in ASJ, we specifically expressed Gα cDNA in ASJ of a Gα triple mutant (*goa-1; gpa-3 gpa-1)*.

While specific expression of *goa-1cDNA*, *gpa-1cDNA* and *gpa-3cDNA* in the AWC sensory neuron of the thermotaxis neural circuit, or in the ASER gustatory neuron, did not significantly rescue the abnormal cold tolerance of the *goa-1; gpa-3 gpa-1* triple mutants (Fig 2B), their abnormal cold tolerance was partially rescued by simultaneously expressing the three Gα cDNAs each driven by its own promoter (Fig 2B, *goa-1*; *gpa-3 gpa-1; Ex[goa-1p*::*goa-1cDNA*, *gpa-1p*::*gpa-1cDNA*, *gpa-3p*::*gpa-3cDNA]*). The abnormal cold tolerance of *goa-1; gpa-3 gpa-1* triple mutants was also partially rescued by expressing *goa-1cDNA*, *gpa-3cDNA* or *gpa-1cDNA* in the ASJ sensory neuron (Fig 2B). This suggests that three Gα proteins, GOA-1, GPA-1 and GPA-3, are involved in cold tolerance in the ASJ temperature-sensing neuron.

Because the abnormality of Gα triple mutants was only partially rescued by simultaneous expression of all three types of Gα cDNAs, driven either by the ASJ specific promoter or by their own promoters, ASJ specific expression of the three cDNAs may be required to almost fully rescue the abnormal cold tolerance of Gα triple mutants. It is also plausible that more complicated machinery, such as alternative splicing isoforms, control this phenomenon.

### Gα signaling is required for the neural response of ASJ to temperature stimuli

The Gα proteins, GOA-1, GPA-1 and GPA-3, work additively in ASJ to regulate cold tolerance. To elucidate in detail the physiological roles of these proteins in temperature sensation by the ASJ sensory neuron, we measured temperature-evoked changes in the intracellular calcium concentration of intact ASJ sensory neurons, using a genetically-encoded calcium indicator, cameleon (Figs 3A–3J and 4A–4J). In *C*. *elegans* neurons, sodium-dependent action potentials do not occur and, instead, voltage-gated calcium channels are rapidly activated [30–36]. Optical recording of calcium concentrations in the ASJ of wild-type animals in this and a previous study revealed that the calcium concentration increased upon warming and decreased upon cooling (Figs 3A, 3B, 4A and 4B) [3].

We introduced the cameleon gene into ASJ neurons of mutant animals defective in either GOA-1, GPA-1 or GPA-3. In *goa-1* or *gpa-3* single mutants, the maximum calcium concentration changes in ASJ were statistically normal when temperature was increased (Fig 3C, 3E and 3H) or decreased (Fig 4C, 4E and 4H). By contrast, calcium concentration changes in ASJ of the *gpa-1* single mutant were slightly abnormal upon warming (Fig 3D and 3H) or cooling (Fig 4D and 4H). This abnormality was not enhanced in the *gpa-3 gpa-1* double mutant (Figs 3F, 3H, 4F and 4H) or the *goa-1; gpa-3 gpa-1* triple mutant (Figs 3G, 3H, 4G and 4H). These observations suggest that at least GPA-1 is involved in temperature signaling in ASJ, and that other unidentified-Gαs or/and other temperature-sensing molecules are potentially involved in primary temperature signaling.

Although the abnormal temperature response of ASJ was observed only in the *gpa-1* mutant (Figs 3D, 3H, 4D and 4H), calcium concentration-changes in ASJ upon cooling after warming were abnormally decreased in *goa-1*, *gpa-1* and *gpa-3* single mutants (Fig 3C–3E, 3I and 3J). However, the *gpa-3 gpa-1* double mutant and the *goa-1; gpa-3 gpa-1* triple mutant showed statistically similar phenotypes to the single mutants, in the calcium imaging (Fig 3F, 3G and 3J). Similar abnormalities were observed in mutant animals defective in guanylyl cyclase (S1A–S1F Fig). Based on these results, we hypothesized that these Gα mutants showed abnormalities in the restoration of the resting calcium concentration in ASJ. If this is the case, Gα mutants may show abnormal neural response upon cooling after warming. We therefore measured the temperature response of ASJ in Gα mutants upon warming after cooling (Fig 4). We found that the calcium concentration changes in ASJ upon warming after cooling were abnormal in the *gpa-1* mutant (Fig 4D, 4I and 4J) as well as in the *gpa-3 gpa-1* double mutant (Fig 4F) and the *goa-1; gpa-3 gpa-1* triple mutant (Fig 4G). These physiological analyses imply that GOA-1, GPA-1 and GPA-3 function in an aspect of restoration of the resting state of calcium concentration upon cooling after warming, whereas only GPA-1 is required for the restoration of calcium status upon warming after cooling. The different physiological roles played by these Gα proteins in temperature signaling may be responsible for their additive genetic effects on cold-tolerance in the multiple mutants of Gα proteins (Fig 1C). Similarly diverse roles are proposed for the guanylyl cyclase proteins (S1G–S1L Fig).

Because the temperature response was not completely diminished in the Gα mutants, other unidentified Gα proteins could be involved in temperature signaling. Alternatively, it is possible that other temperature-sensing molecules, such as TRP channels and HSPs encoded by many genes are also involved in temperature signaling.

### Abnormality in PDE induces inactivation of ASJ temperature-sensing neuron

Phosphodiesterase (PDE) is well known to function as a negative regulator of intracellular calcium influx in sensory signaling, by hydrolysis of cGMP; conversely, up-regulation of cGMP is mediated by GC [11]. We previously reported that PDE is required for cold tolerance of *C*. *elegans*, and that *pde* mutants showed abnormal enhancement of cold tolerance [3]. This previous result seems to be inconsistent with a model in which PDE acts as a negative regulator of calcium concentration changes in ASJ temperature signaling; *pde* mutation would be expected to cause hyperactivation of ASJ, which would induce an abnormal decrease of cold tolerance. We therefore measured calcium influx in ASJ of *pde* mutants under temperature stimuli (Figs 5A–5I and 6A–6I).

We found that calcium concentration changes were decreased upon warming in ASJ of *pde-2* (Fig 5B and 5G) or *pde-3* mutants (Fig 5C and 5G). A similar abnormality was observed in the *pde-1 pde-5; pde-2* triple mutant. This abnormality was enhanced by *pde-3* mutation, as observed in the *pde-1 pde-5; pde-3; pde-2* quadruple mutant (Fig 5E, 5F and 5G). Additionally, the *pde-1 pde-5; pde-3; pde-2* quadruple mutant showed an abnormally decreased calcium concentration change in ASJ upon cooling (Fig 5F, 5H and 5I). As previously reported, a strong calcium influx into vertebrate photosensory neurons negatively regulates GC, which causes a decrease in calcium influx in the neuron [21,22]. Based on these previous studies and the present study, we propose a model in which strong calcium influx into ASJ of *pde* mutants negatively regulates sensory signaling (e.g., via GC), resulting in inactivation of the sensory neuron; this is consistent with observations on the photoreceptor cells of vertebrate.

The calcium concentration in ASJ was increased upon warming, and decreased upon cooling in wild-type animals. In *pde-3* mutants, the decrement of calcium concentration under cooling was weaker than that in the wild type (Fig 6C and 6G). In contrast, in *pde-5* mutants, the increment of calcium concentration under warming was stronger than that in the wild type (Fig 5D and 5G). These observations suggest that PDE-3 acts as a negative regulator in the response to cooling stimuli, and that PDE-5 acts as a negative regulator in the response to warming stimuli. This scheme is consistent with the molecular function of PDE, which is a negative regulator of intracellular calcium influx in sensory signaling in many animals [11]. To investigate whether PDE-3 and PDE-5 are downstream of G protein in ASJ temperature signaling, we carried out genetic epistasis analysis (Fig 7). Because the *gpa-1* mutant showed abnormal calcium concentration changes in ASJ upon both warming and cooling stimuli (Figs 3D and 4D), we constructed *pde-5; gpa-1* and *pde-3; gpa-1* double mutants and measured temperature responses of ASJ by calcium imaging. The abnormal temperature response of ASJ in the *pde-5; gpa-1* mutant was similar to that of the *pde-5* single mutant when the temperature was increased (Fig 7A). Also, the abnormal temperature response of ASJ in the *pde-3; gpa-1* mutant was similar to that of the *pde-3* mutant when the temperature decreased (Fig 7E). These results indicate that these PDE mutations are epistatic to G protein mutation, which is consistent with a molecular pathway in which PDEs are downstream of G-protein.

Previously, it was reported that the PDE mutants used in this study showed abnormal ASJ neuronal responses at the electrophysiological level to light stimuli, suggesting that these PDEs are functional in the ASJ sensory neuron [19]. However, although these PDEs may function in ASJ sensory signaling, it is also possible that they operate in other unidentified-thermosensory neuron(s) involved in cold tolerance.

### Shared and separate molecular pathways underlie temperature sensation and phototransduction in ASJ

Analysis of cold tolerance of animals defective in G-protein temperature signaling of ASJ demonstrated that multiple Gα proteins additively regulate cold tolerance. Calcium imaging of animals defective in temperature signaling in ASJ showed that multiple Gα proteins, guanylyl cyclases and phosphodiesterases have physiologically diverse roles in temperature signaling, which are possibly linked to genetic interactions in relation to cold tolerance.

**Fig 8:**
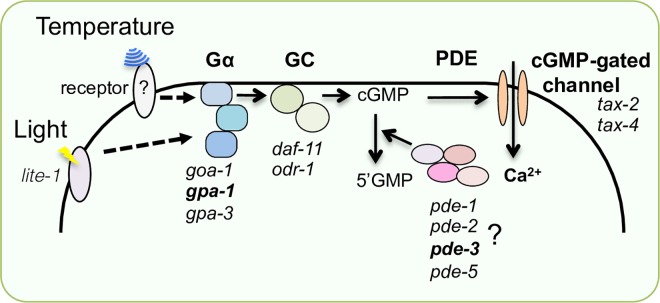
A molecular model for light and temperature signaling in ASJ sensory neuron, which controls cold tolerance. Some molecules are thought to be common, and some specific, to the temperature- and light-signaling pathways of ASJ. Gene name shown in bold indicate molecules specific to temperature signaling. Because the temperature response was not completely extinguished in the mutants in this study, unidentified signaling molecules such as Gα, GC and PDE may also be required for temperature signaling. The temperature receptor has not been identified.

Based on these molecular physiological data, we propose differences and commonalities in the molecular components involved in sensing temperature and light (Fig 8). In phototransduction, a light stimulus is received by light receptor LITE-1, which activates the Gα proteins GOA-1 and GPA-3 additively, regulating GCs, DAF-11 and ODR-1. These GCs produce cGMP as a second messenger, and cGMP-dependent cation channels composed of TAX-2 and TAX-4 are crucial for the activation of ASJ (Fig 8). Three PDEs (PDE-1, PDE-2 and PDE-5) additively hydrolyze cGMP to 5’GMP, and neuronal activity is decreased. In temperature sensation, temperature is received by an unidentified receptor, which activates the Gα subunits GOA-1, GPA-1, GPA-3 and an additional unidentified Gα, which additively regulates the GC, DAF-11, ODR-1, and another unidentified GC (Fig 8). These GCs produce cGMP, which activates TAX-2 and TAX-4. Multiple PDEs (PDE-1, PDE-2. PDE-3 and PDE-5) additively hydrolyze cGMP to 5′GMP. The commonalities and differences in the molecules involved in light and temperature signaling of the ASJ sensory neuron are summarized in Fig 8. It is also possible that temperature signaling is accomplished by a more complicated molecular physiological system, although we describe here a simple and plausible molecular pathway.

The complex tissue network involved in cold tolerance means that a small impairment in ASJ signaling might cause a large abnormality in cold tolerance. As reported previously, cold tolerance is mediated through multiple steps, including temperature sensation and insulin secretion in a sensory neuron, insulin signaling and gene expression in the intestine, and a feedback mechanism from sperm to the sensory neuron [37]. Because orchestration of these multiple steps is important for accomplishing normal cold tolerance, a partial defect in signaling within the network, such as temperature reception in the sensory neuron, could strongly affect cold tolerance. In the current study, cold tolerance was induced on a timescale of hours, whereas calcium imaging was studied over minutes. Although Gα mutants showed different abnormalities in calcium imaging under short-term temperature stimuli, it is possible that Gα mutants would show greater phenotypic differences in the calcium-imaging analysis if studied over a longer time frame [3,38].

The decrement of calcium concentration in ASJ in the *pde-3* mutant under cooling was smaller than in the wild type, but this abnormality was not observed in other *pde* mutants (Fig 6). In contrast, the increment of calcium concentration under warming was larger than that of the wild type only in the *pde-5* mutant (Fig 5). It is probable that the functioning molecules in G-protein-mediated temperature signaling are partially different between cooling and warming stimuli. This speculation is consistent with the observation that mutations in guanylyl cyclase (DAF-11 and ODR-1) impaired the temperature response in ASJ on warming, but not on cooling (S1 Fig).

The molecular mechanisms underlying sensory signaling are mainly conserved from *C*. *elegans* to humans. Thus, the system described in this study provides useful information for studying temperature sensation in other animals.

## Supporting Information

S1 Fig Calcium concentration changes in ASJ of GC mutants with warming or cooling stimuli. (A–C) Calcium imaging of ASJ from wild-type and GC mutants cultivated at 20°C. The transgene of *trx-1p*::*yc3*.*60* was introduced into each GC mutant and we measured relative calcium concentrations under warming stimuli, as in the wild-type experiment (the pale blue line indicates calcium concentration changes in the wild-type animals shown in Fig 3B; these experiments were performed simultaneously; *n* = 17–23). Temperature changes during the experiment are indicated in the bottom chart. (D) The bar chart shows the average ratio changes during 5 s before the maximum point to 5 s after the maximum point in the experiments shown in Figs 3B and S1A–C. (E) The bar chart shows the average ratio changes around the minimum point during 10 s from 280 to 290 s in the experiments shown in Figs 3B and S1A–C. (F) The bar chart shows the average ratio changes of the difference value between maximum and minimum points in the experiments shown in Figs 3B and S1A–C. Colors used in bar graphs D–F are the same as those used for the corresponding response curves in A–C. (G–I) We measured relative calcium concentration under cooling stimuli as in the wild-type experiment (the pale blue line indicates calcium concentration changings in the wild-type animals shown in Fig 4B; these experiments were performed simultaneously; *n* = 15–21). Temperature changes during the experiment are indicated in the bottom chart. (J) The bar chart shows the average ratio changes during 5 s before the maximum point to 5 s after the maximum point of the experiments shown in Figs 4B and S1G–I. (K) The bar chart shows the average ratio changes from around the minimum point during 10 s from 280 to 290 s of the experiments shown in Figs 4B and S1G–I. (L) The bar chart shows the average ratio changes of the difference value between maximum and minimum points of the experiments shown in Figs 4B and S1G–I. Error bars indicate SEM (A–L). Analysis of variance followed by Dunnett’s *post-hoc* test was used for multiple comparisons (D–F, J–L, compared with the wild type). *P < 0.05; **P < 0.01. Colors used for the bar graphs J–L are the same as those used for the corresponding response curves in G–I.
(TIF)

S1 Text Discussion on temperature responses of ASJ sensory neruons in variety of G protein signaling mutants. (DOCX)
