# Supplementary figures and images for: Diverse Regulation of Temperature Sensation by Trimeric G-Protein Signaling in Caenorhabditis elegans
Source: PLoS One. 2016 Oct 27;11(10):e0165518. doi: 10.1371/journal.pone.0165518 (PMC5082853; doi:10.1371/journal.pone.0165518)

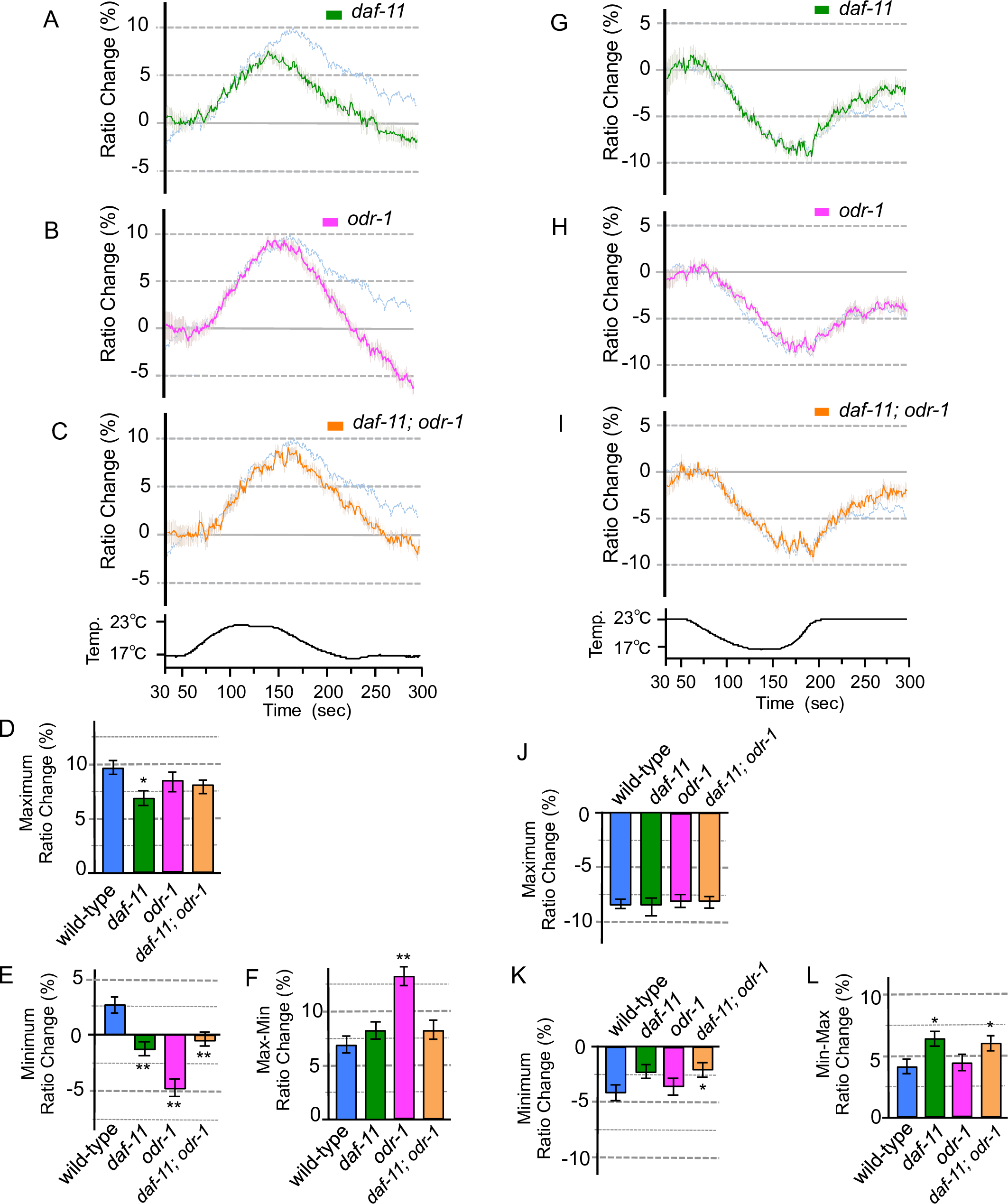

Supplement: S1 Fig — (A–C) Calcium imaging of ASJ from wild-type and GC mutants cultivated at 20°C. The transgene of trx-1p::yc3.60 was introduced into each GC mutant and we measured relative calcium concentrations under warming stimuli, as in the wild-type experiment (the pale blue line indicates calcium concentration changes in the wild-type animals shown in Fig 3B; these experiments were performed simultaneously; n = 17–23). Temperature changes during the experiment are indicated in the bottom chart. (D) The bar chart shows the average ratio changes during 5 s before the maximum point to 5 s after the maximum point in the experiments shown in Figs 3B and S1A–C. (E) The bar chart shows the average ratio changes around the minimum point during 10 s from 280 to 290 s in the experiments shown in Figs 3B and S1A–C. (F) The bar chart shows the average ratio changes of the difference value between maximum and minimum points in the experiments shown in Figs 3B and S1A–C. Colors used in bar graphs D–F are the same as those used for the corresponding response curves in A–C. (G–I) We measured relative calcium concentration under cooling stimuli as in the wild-type experiment (the pale blue line indicates calcium concentration changings in the wild-type animals shown in Fig 4B; these experiments were performed simultaneously; n = 15–21). Temperature changes during the experiment are indicated in the bottom chart. (J) The bar chart shows the average ratio changes during 5 s before the maximum point to 5 s after the maximum point of the experiments shown in Figs 4B and S1G–I. (K) The bar chart shows the average ratio changes from around the minimum point during 10 s from 280 to 290 s of the experiments shown in Figs 4B and S1G–I. (L) The bar chart shows the average ratio changes of the difference value between maximum and minimum points of the experiments shown in Figs 4B and S1G–I. Error bars indicate SEM (A–L). Analysis of variance followed by Dunnett’s post-hoc test was used for multiple compar [file pone.0165518.s001.tif]
