# Supplementary material for: Diverse Regulation of Temperature Sensation by Trimeric G-Protein Signaling in Caenorhabditis elegans
Source: PLoS One. 2016 Oct 27;11(10):e0165518. doi: 10.1371/journal.pone.0165518 (PMC5082853; doi:10.1371/journal.pone.0165518)
Supplement: S1 Text — (DOCX) [file pone.0165518.s002.docx]

**Discussion on temperature responses of ASJ sensory neruons in variety of G protein signaling mutants.**

We analyzed ratio changes of yellow cameleon YC3.60 to compare neuronal activity of the ASJ temperature-sensing neuron between wild-type and various mutants defective in temperature signaling. To help interpret the biological meaning of calcium traces in the calcium-imaging analysis, we calculated three parameters, Maximum (Max), Minimum (Min), and Max−Min (or Min−Max), from each trace. Max refers to the value of the calcium response to the first temperature stimulus (warming or cooling), and Min refers to terminal point of the calcium response after return to the initial temperature (in both cases, the calcium concentration typically returned to a lower value than that initially). Max−Min (in the case of warming stimuli) or Min−Max (in the case of cooling stimuli) refers to the difference between the values of calcium responses to the first and the second temperature stimuli (warming followed by cooling or cooling followed by warming). When Min is abnormal, these Max−Min or Min−Max values reflect both the value of the calcium response to the second temperature change and/or an abnormal return to the resting state. Using these parameters, two representative phenotypes in *goa-1* and *pde-2* could be identified. For example:

In 17°C ->23°C ->17°C stimuli, the *goa-1* mutant showed normal Max, abnormal Min and abnormal Max−Min values (Fig 3C, I, J). This implies that *goa-1* showed a normal response to warming stimuli and an abnormal response to cooling stimuli, or an abnormal return to the resting calcium concentration status. In the case of the opposite 23°C->17°C ->23°C stimuli, the *goa-1* mutant did not show any abnormalities (Fig 4 C, H–J), implying that *goa-1* showed a normal response to cooling stimuli. Together, these observations suggest that the *goa-1* mutant was abnormal in returning calcium concentration to the resting state after warming stimuli.

In the case of 17°C ->23°C ->17°C stimuli, the *pde-2* mutant showed abnormal Max and Min values (Fig 5B, G, H), but Max−Min was normal (Fig 5B, I). This implies that *pde-2* showed an abnormal response to warming stimuli, and a normal response to cooling stimuli, resulting in an abnormal return of the calcium concentration to the resting state, despite a normal cooling response. With the opposite 23°C ->17°C ->23°C stimuli, *pde-2* showed normal Max, but abnormal Min and Min−Max values (Fig 6B, G–I). These results are consistent with the results of the 17°C ->23°C ->17°C stimuli in which *pde-2* showed an abnormal response to warming stimuli and a normal response to cooling stimuli, abnormally returning calcium concentration to the resting state.
